# Editorial: Otitis media

**DOI:** 10.3389/fcimb.2022.1063153

**Published:** 2022-11-25

**Authors:** Kevin M. Mason, Robyn L. Marsh, Stephen I. Pelton, Eric T. Harvill

**Affiliations:** ^1^ Center for Microbial Pathogenesis, The Abigail Wexner Research Institute at Nationwide Children’s Hospital, Columbus, OH, United States; ^2^ Infectious Diseases Institute, The Ohio State University, Columbus, OH, United States; ^3^ Child Health Division, Menzies School of Health Research, Charles Darwin University, Darwin, NT, Australia; ^4^ Boston University Chobanian & Avedisian School of Medicine, Boston, MA, United States; ^5^ Department of Pediatrics, Boston Medical Center, Boston, MA, United States; ^6^ Department of Infectious Diseases, College of Veterinary Medicine, University of Georgia, Athens, GA, United States

**Keywords:** otitis media, pathogenesis, diagnosis, hearing, deafness, immunity, vaccine, childhood disease

Middle ear infection remains a significant health concern with 80 percent of young children, primarily between the ages of 6 and 24 months, experiencing at least one otitis media (OM) episode and many suffering multiple recurrences each year (Pichichero, 2013; Mittal et al., 2015; Rosenfeld et al., 2016; Tong et al., 2018). Recurrent acute OM is one of the most common indications for tympanostomy tube placement surgery and antibiotic prescription, with staggering associated treatment costs and impacts on emerging resistance (Pelton et al., 2013). Further burden arises from OM-associated hearing loss that impacts behavior, language development and educational outcomes (Monasta et al., 2012; Schilder et al., 2016; Homoe et al., 2019), with such impacts compounded among global populations with high chronic suppurative OM burden (Leach et al., 2020). Antibiotics remain the preferred treatment to sterilize the bacterial component of disease and abort the inflammatory process in the middle ear; however, infections often persist, resulting in ongoing inflammation and hearing impacts. Despite the vast global disease burden, much remains unknown about OM pathogenesis.

This Research Topic provides insight into the current state of the field, highlighting recent research targeted at critical gaps in understanding of the microbial epidemiology and wider pathobiology of OM. Topics covered extend from ongoing work to define temporal and geographic variability in OM microbiology, through to human and animal model studies exploring host and microbial factors underlying the disease, and implementation of modern computational approaches for improving OM diagnostics.

## Temporal and geographic impacts on OM microbial epidemiology

During the COVID-19 pandemic, decreased acute OM presentations were reported for several populations [reviewed in (Marom et al.)]. In a Dutch primary care cohort study (Hullegie et al.), episodes of OM were shown to decrease during the pandemic, despite similar antibiotic prescription rates for acute OM pre- and post-pandemic. A decrease in OM episodes was likely the result of implementation of quarantine measures to control the spread of the COVID-19 pandemic. This observation is a reminder of the potential for public health interventions to disrupt respiratory pathogen transmission and reduce OM disease burden; an observation of particular importance where overcrowded housing is associated with increased OM burden (DeLacy et al., 2020). The direct impact of SARS-CoV2 on middle ear microbiology is not known. As reviewed by Marom et al., a small number of studies have reported SARS-CoV2 detection from middle ear specimens; however, our understanding of this virus in OM remains limited.

Pneumococcal conjugate vaccines impact the population biology of *Streptococcus pneumoniae*; an important otopathogen. Although vaccination has reduced the incidence of disease due to pneumococcal vaccine serotypes, the emergence of replacement serotypes and increased incidence of other otopathogens warrants further investigation. This important point is highlighted in work by Fuji et al. demonstrating the emergence of acute OM-associated *S. pneumoniae* serotype 35B genotypes since 2015. Genomic changes among strains recovered after 2015 - including single nucleotide polymorphisms (SNPs) found in metal binding, ABC transporters and DNA replication genes - may be associated with increased virulence (Fuji et al.).

OM microbiology varies across populations, with population-level effects known to impact key drivers of disease (Sakulchit and Goldman, 2017; DeLacy et al., 2020; Dagan et al., 2021). Geographic variation in OM pathobiology was highlighted by Australian studies examining differences among populations from distinct climatic zones and with variable socio-economic advantage. Clark et al. examined natural IgG antibody titers and avidity to three putative nontypeable *Haemophilus influenzae* (NTHI) vaccine antigens among children living in urban and remote Western Australia. The investigators showed that Australian Aboriginal otitis-prone children had lower serum IgG titers to two of the vaccine candidates compared to that of non-Aboriginal otitis-prone children and non-otitis prone children, potentially indicating a blunting of the immune response among children with early-onset OM (Thornton et al., 2017; Renz et al., 2018; Leach et al., 1994). The results also suggest possible benefits from immunization to boost antibodies against NTHI proteins (Clark et al.). In a second study, Ngo et al. showed NTHI and rhinovirus were the predominant otopathogens within the upper respiratory tract (URT) of children with and without OM from peri-urban and urban South-East Queensland (Ngo et al.). The presence of bacterial otopathogens in the middle ear was more predictive of concurrent URT infection than those associated with virus (Ngo et al.). As with earlier studies (Hall-Stoodley et al., 2006), PCR-based otopathogen detection was more sensitive than culture, potentially related to intracellular and biofilm-associated infections.

## Relationships between host genomics and the airway microbiome in OM

Genetic markers of increased OM risk are poorly understood, but candidate genes are emerging. In the study by Elling et al., variants in alpha-1,2-fucosyltransferase (Fut2) were associated with an increased susceptibility to OM, potentially through a shift in nasopharyngeal or middle ear microbiota. The variant was associated with transcriptional changes in processes related to response to infection, increased pathobiont load in the middle ear and decreased nasopharyngeal commensals.

Microbiome shifts were also observed among patients with cholesteatoma. Frank et al. collected middle ear samples from patients undergoing tympanomastoidectomy for chronic OM and showed that factors such as quinolone use and cholesteatoma diagnosis had a substantial effect on middle ear microbiota composition.

## Variable gene expression among OM pathogens

The complexity of microbial factors underlying OM are compounded by phenotypic adaption of otopathogens to different airway niches. Janouskova et al. reviewed gene regulation mechanisms that are well-orchestrated among otopathogens during OM in both experimental and in clinical settings. With a focus on phase variable and quorum sensing systems, the authors examined the ability of otopathogens to adapt inside the host to benefit survival and persistence. Whether this translates to increased opportunity for transmission and spread within and between populations remains an open question.

## Immune responses in OM

Advances in understanding middle ear immunology provide opportunities to engineer and test more targeted and effective OM treatments. A timely review by Massa et al., examines innate immunity in the middle ear in response to bacterial, viral or polymicrobial insult, demonstrating that localized inflammatory responses can persist even after clearance of the bacteria and virus from the middle ear. Otitis-prone children display a strong immune response suggesting that immunomodulatory therapeutics could be of benefit in these patients.

Multiple studies in this collection used *in vitro* models to investigate middle ear responses to infection, with several studies focused on middle ear hyperplasia and leukocyte infiltration – hallmarks of OM pathogenesis. Leichtle et al. showed in patients with chronic disease, persistent mucosal hyperplasia resulted in increased expression of inflammatory and apoptotic genes. Therapeutic approaches to dampen these responses could lessen the consequences of chronic disease. The use of single-cell RNA sequencing identified middle ear cells expressing genes associated with hyperplasia. Sakamoto et al. expanded upon these observations to show that a synthetic analog of viral double-stranded RNA (which simulates a middle ear viral infection) induced heparin-binding epidermal growth factor (HB-EGF) expression in middle ear epithelial, stromal and endothelial cells. Single-cell RNA sequencing also revealed that the eicosanoid leukotriene B4 (LTB4), an arachidonic acid metabolite, mediated leukocyte recruitment during OM (Heo et al.). LTB4 inhibition decreased mucosal hyperplasia and leukocyte infiltration (Heo et al.), suggesting potential therapeutic utility of LTB4 receptor antagonists in OM.

Animal models were also used to investigate how environmental exposures influence host responses. In an LPS-induced model of acute OM, Kim et al. demonstrated that mice treated with LPS and exposed to diesel exhaust particles (DEP) demonstrated mucosal cell hyperplasia and increased expression of TNF-alpha and IL1-beta. Pre-exposure to DEP increased inflammation and lymphangiogenesis.

## Emerging OM model systems

Two studies reported the development and use of novel animal models that may allow more detailed mechanistic understanding of middle ear immune functions. Dewan et al. observed that *Bordetella bronchiseptica*, a respiratory commensal and pathogen of mice, ascends the Eustachian tube to colonize the middle ears, providing a model of naturally occurring acute OM. The investigators showed that mice lacking T and B cells failed to clear infection and that adoptive transfer of antibodies cleared lung infection but only partially cleared ear infection.

In a second study, Ma et al. demonstrated that small numbers of *Bordetella pseudohinzii* introduced into the nasopharynx can ascend the Eustachian tube and colonize and persist in the middle ear to model chronic infection. Secretion of a novel pertussis toxin-like factor was associated with prolonged persistence of middle ear pathology leading to hearing loss.

## New diagnostic technologies

Data science approaches are increasingly leveraged to extract nuanced understanding of complex biological systems. Large, well-curated datasets are likely to be essential to realizing meaningful outcomes from studies using computational approaches, including artificial intelligence (AI). Current efforts to prepare large scale OM datasets include the Australian BIGDATA study reported by Beissbarth et al. – a compilation of 11 randomized controlled trials, 4 cohort studies, 8 surveys in over 30 remote communities and 5 surveys in urban childcare centers - that is expected to provide powerful insight into OM trends, otopathogen carriage rates and the impact of three sequential pneumococcal conjugate vaccines on pneumococcal serotype replacement among an Australian population at high-risk of OM.

Data science-based approaches are also supporting development of new diagnostic applications. Duan et al. provide an in-depth discussion of this issue and demonstrate a deep learning framework for deriving imaging-based diagnostic results of temporal bone diseases, cholesteatoma, middle ear inflammation and Langerhans cell histiocytosis that outperformed clinical expert-based diagnoses.

There is also vast opportunity for digital technologies to support telemedicine-based OM clinical care, as done during the COVID pandemic (Marom et al.). Though not without challenges, telemedicine consultations are also expected to increase in the coming years, with high potential to enable access to care for populations in geographically remote areas (Kokesh et al., 2011).

## Conclusions

Over the past decade we have witnessed the advent of new advanced genomic methodologies, bioinformatics, coordinated multi-systems data analysis, microbiome investigations and the increased use of telemedicine and artificial intelligence platforms for diagnosis of disease. We are now witnessing the impact of these advances in the study of otitis media. The collection of articles in this research topic encompasses the breadth of work being done to advance interventions in public health, clinical diagnosis, and therapeutic development; work that is critically needed to realize our collective goal of reducing, and ultimately eliminating, the global OM disease burden.

## Author contributions

All authors listed have made a substantial, direct, and intellectual contribution to the work and approved it for publication.

## Funding

This work was supported by NIH grants AI139519, AI164077 and DC013313 (KM), Al and Val Rosenstrauss Fellowship, provided by the Rebecca L Cooper Foundation, Australia (RM), NIH grant DC015050 (SP), NIH grants AI159347, AI142678 and DC018496 (EH).

## Conflict of interest

The authors declare that the research was conducted in the absence of any commercial or financial relationships that could be construed as a potential conflict of interest.

## Publisher’s note

All claims expressed in this article are solely those of the authors and do not necessarily represent those of their affiliated organizations, or those of the publisher, the editors and the reviewers. Any product that may be evaluated in this article, or claim that may be made by its manufacturer, is not guaranteed or endorsed by the publisher.
